# Outcomes after Perioperative Transient Ischemic Attack Following Cardiac Surgery

**DOI:** 10.3390/jcdd11010027

**Published:** 2024-01-17

**Authors:** Urvish Jain, Bhav Jain, James Brown, Ibrahim B. Sultan, Floyd Thoma, Katherine M. Anetakis, Jeffrey R. Balzer, Kathirvel Subramaniam, Sarah Yousef, Yisi Wang, Raul Nogueira, Parthasarathy D. Thirumala

**Affiliations:** 1 School of Medicine, University of Pittsburgh, Pittsburgh, PA 15213, USA; urj2@pitt.edu; 2 School of Medicine, Stanford University, Stanford, CA 94305, USA; bhavjain@stanford.edu; 3 Department of Cardiothoracic Surgery, University of Pittsburgh Medical Center, Pittsburgh, PA 15213, USA; brownja7@upmc.edu (J.B.); yousef.sarah@medstudent.pitt.edu (S.Y.); wangy21@upmc.edu (Y.W.); 4 Department of Neurological Surgery, University of Pittsburgh Medical Center, Pittsburgh, PA 15213, USA; katherine.anetakis@chp.edu (K.M.A.); jeb23@pitt.edu (J.R.B.); thirumalapd@upmc.edu (P.D.T.); 5 Department of Anesthesiology and Perioperative Medicine, University of Pittsburgh Medical Center, Pittsburgh, PA 15213, USA; kas338@pitt.edu; 6 Department of Neurology, University of Pittsburgh Medical Center, Pittsburgh, PA 15213, USA; rgn7@pitt.edu

**Keywords:** cardiac surgery, transient ischemic attack, perioperative transient ischemic attack

## Abstract

Perioperative transient ischemic attacks (PTIAs) are associated with significantly increased rates of postoperative complications such as low cardiac output, atrial fibrillation, and significantly higher mortality in cardiac procedures. The current literature on PTIAs is sparse and understudied. Therefore, we aim to understand the effects of PTIA on hospital utilization, readmission, and morbidity. Using data on all the cardiac procedures at the University of Pittsburgh Medical Center from 2011 to 2019, fine and gray analysis was performed to identify whether PTIAs and covariables correlate with increased hospital utilization, stroke, all-cause readmission, Major Adverse Cardiac and Cerebrovascular Events (MACCE), MI, and all-cause mortality. Logistic regression for longer hospitalization showed that PTIA (HR: 2.199 [95% CI: 1.416–3.416] increased utilization rates. Fine and gray modeling indicated that PTIA (HR: 1.444 [95% CI: 1.096–1.902], *p* < 0.01) increased the rates of follow-up all-cause readmission. However, PTIA (HR: 1.643 [95% CI: 0.913–2.956] was not statistically significant for stroke readmission modeling. Multivariate modeling for MACCE events within 30 days of surgery (HR: 0.524 [95% CI: 0.171–1.605], *p* > 0.25) and anytime during the follow-up period (HR: 1.116 [95% CI: 0.825–1.509], *p* > 0.45) showed no significant correlation with PTIA. As a result of PTIA’s significant burden on the healthcare system due to increased utilization, it is critical to better define and recognize PTIA for timely management to improve perioperative outcomes.

## 1. Introduction

Currently, the consequences of perioperative transient ischemic attacks (PTIAs) are understudied, and relatively little literature exists on both short- and long-term patient recovery. PTIAs are associated with significantly increased rates of low cardiac output, atrial fibrillation, and significantly higher mortality in cardiac procedures [1]. While relatively uncommon in low-risk surgical procedures, the current literature on PTIAs is sparse. In 2020, the Society for Thoracic Surgery (STS) removed PTIA from their operative discharge survey, further reducing the data on and research into PTIAs.

In non-operative settings, TIA has been shown to increase the risk of recurrent TIA, stroke, cardiovascular events, and mortality [2]. In some cases, TIA has an imaging correlation with and is labeled as stroke for appropriate evaluation and management. This has led to changes in the definition of TIA in non-operative settings, and clinical trials like ABCD and ABCD2 to evaluate effective management [3]. PTIA and PTIA-like symptoms can be due to TIA, infections, or stroke/seizure/any metabolic derangement causing altered mental status or cardiac complications, which makes PTIA often underreported or missed. Additionally, the neurological changes may be subtle or brief, further complicating estimation of its incidence. It is not always evaluated after vascular surgery even though it has a high incidence, in which case it is silent in imaging tools [4].

Therefore, to understand the effects of PTIA, we used our institution’s STS data set to evaluate whether PTIA (1) increases the length of stay in the hospital and ICU, (2) increases readmission from stroke and all causes, and (3) increases Major Adverse Cardiac and Cerebrovascular Events (MACCE), stroke, MI, and all-cause mortality within 30 days and during the follow-up period. We expect these results will be helpful to the surgical and stroke community in understanding the impact of perioperative TIAs and improving awareness and recognition. We also expect these results to be helpful in designing therapeutic interventions for the effective management of perioperative TIAs.

## 2. Patients and Methods

### 2.1. Study Population

**Figure 1:**
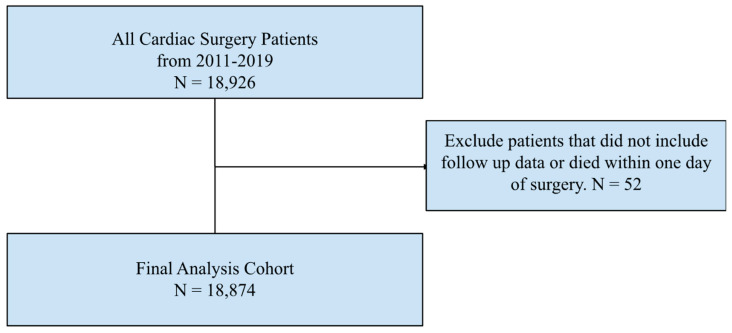
Study Design and Exclusion Criteria.

Using data from all cardiothoracic surgeries (Aortic Root, CABG and AV, CABG and MV, CABG and PV, CABG and TV, Double Valve, Isolated AV, Isolated CABG, Isolated MV, Isolated PV, Isolated TV, TAVR, Triple Valve, Other) at the University of Pittsburgh Medical Center (UPMC), we identified 18,926 total index cases to analyze from 2011 through 2019. This study period was chosen as the STS removed TIA from follow-up surveys in 2020. Cases that did not include follow-up data or with patients that died within one day of surgery were excluded, leaving 18,874 total cases. (Figure 1) All ischemic, hemorrhagic, and undetermined strokes were included in the patient population. All urgent, emergent, and elective cases were included. The operative data were retrospectively obtained from a prospectively maintained cardiac surgical database. This study was approved by the Institutional Review Board of the University of Pittsburgh on 17 April 2019 (STUDY18120143), with written consent being waived.

### 2.2. Definitions

Transient Ischemic Attack: Defined based on the STS guidelines of a patient having a history of loss of neurological function that was abrupt in onset but with a complete return of functionality within 24 h, presumed to be of vascular etiology; this does not include neurological disease processes such as metabolic and/or anoxic ischemic encephalopathy [5]. The TIA data were further adjusted in the clinical data extraction.

Longer Length of Stay: ICU length of stay was considered longer if it was longer than 3 days. Hospital length of stay was considered longer if it was longer than 14 days [6].

Readmission: Defined based on the STS definition of whether a patient was readmitted to any hospital within 30 days of discharge because of any cause related to the previous operation [5].

MACCE: Major Adverse Cardiac and Cerebrovascular Events defined based on the previous literature as death, stroke, subsequent revascularization, or hospitalization for MI and heart failure [7,8].

### 2.3. Statistical Analysis:

The primary outcome measurements in this study were hospital utilization based on longer length of stay, stroke-related or all-cause readmission, and a compound morbidity outcome of six variables defined by the Society of Thoracic Surgeons. These outcomes included (1) in-hospital death, (2) acute postoperative myocardial infarction, (3) neurologic morbidity, including focal or global neurologic deficits or death without awakening, (4) serious infection morbidity consisting of sepsis syndrome or septic shock, (5) new-onset renal failure requiring dialysis, and (6) postoperative ventilatory support exceeding 72 h. These outcomes were compared to covariables in the model that were selected by physicians. These covariables included age, sex, diabetes, prior heart failure, cardiovascular accidents, and PTIA. The categorical variables were presented by count and percentage; the continuous variables were presented by Mean ± S.D. or Median with IQR. When applicable, Chi-square or Fisher’s exact tests were used for categorical variable comparison. Normality was assessed using the Kolmogorov–Smirnov (K–S) test, Student’s *t*-test, or the nonparametric Mann–Whitney U test for continuous variables when appropriate. Survival was compared using Kaplan–Meier analysis (log rank test). The proportional hazard assumption was tested using Schoenfeld residuals. For the readmission models, competing risk analysis with a fine and gray model was used, and death was the competing risk event. Follow-up started when surgery was performed; for readmission events, follow-up started at discharge from hospital. The all-cause mortality data were verified against the U.S. Social Security Death Index database. If last follow-up dates were missing, the case was censored on the date we pulled out the data.

## 3. Results

### 3.1. Baseline Characteristics

Of the 18,874 total index cases between 2011 and 2019, 1194 (6.33%) were Aortic Root, 1508 (7.99%) were CABG and AV, 615 (3.26%) were CABG and MV, 2 (0.01%) were CABG and PV, 26 (0.14%) were CABG and TV, 1055 (5.59%) were Double Valve, 2318 (12.28%) were Isolated AV, 7725 (40.93%) were Isolated CABG, 1305 (6.91%) were Isolated MV, 15 (0.08%) were Isolated PV, 111 (0.59%) were Isolated TV, 1575 (8.34%) were TAVR, 96 (0.51%) were Triple Valve, and 1329 (7.04%) were classified as Other.

Furthermore, 82 of the cases were identified as PTIA, giving a rate of 0.43% in the analysis cohort. A total of 6500 (34.4%) patients were female, and the median age of all patients was 68 (q1–q3, 59–76). Equally, 17,474 (92.5%) were white, 910 (4.8%) were black, and 490 (2.6%) were other races. The main comorbidities in the population included 7131 (37.7%) patients that had diabetes, 5753 (30.48%) patients that had previous heart failure, 15,808 (83.7%) patients that had hypertension, and 7857 (41.6%) that had previous myocardial infarctions. (Table 1).

### 3.2. PTIA and Hospital Utilization

A total of 6502 (34.5%) patients had extended lengths of stay (patient in ICU for over 3 days or total length of stay over 14 days) with the median stay at 8 (q1–q3, 5–12) days after surgery. Logistic regression for longer hospitalization showed that PTIA (HR: 2.199 [95% CI: 1.416–3.416], *p* < 0.001), being female (HR: 1.216 [95% CI: 1.141–1.296], *p* < 0.001), diabetes (HR: 1.267 [95% CI: 1.191–1.349], *p* < 0.001), prior heart failure (HR: 1.421 [95% CI: 1.330–1.518], *p* < 0.0001), and cerebrovascular accidents (HR: 1.695 [95% CI: 1.534–1.873], *p* < 0.0001) were all associated with increased utilization rates. Surprisingly, higher age (HR: 0.993 [95% CI: 0.990–0.995], *p* < 0.0001) was negatively associated with longer hospitalization (Table 2).

### 3.3. PTIA and Risk of Readmission

A total of 1636 (8.7%) of the analysis cohort had stroke readmission and 10,437 (55.3%) had all cause readmission in the follow-up period. The fine and gray modeling indicated that diabetes (HR: 1.205 [95% CI: 1.091–1.330], *p* < 0.001) and cerebrovascular accidents (HR: 1.772 [95% CI: 1.545–2.033], *p* < 0.001) were key indicators for follow-up stroke readmission. PTIA (HR: 1.643 [95% CI: 0.913–2.956], *p* < 0.10), age (HR: 1.003 [95% CI: 0.999–1.007], *p* < 0.15), being female (HR: 1.018 [95% CI: 0.919–1.128], *p* < 0.75), and prior heart failure (HR: 1.065 [95% CI: 0.956–1.186], *p* < 0.30) were not statistically significant to the stroke readmission modeling.

The fine and gray modeling indicated that PTIA (HR: 1.444 [95% CI: 1.096–1.902], *p* < 0.01), being female (HR: 1.077 [95% CI: 1.034–1.122], *p* < 0.0001), diabetes (HR: 1.248 [95% CI: 1.200–1.298], *p* < 0.0001), prior heart failure (HR: 1.279 [95% CI: 1.226–1.334], *p* < 0.0001), and previous cerebrovascular accidents (HR: 1.196 [95% CI: 1.121–1.77], *p* < 0.0001) were all causes of increased rates of follow-up all-cause readmission. The results for age (HR: 1.001 [95% CI: 0.999–1.002], *p* < 0.45) and all-cause readmission were not statistically significant (Table 3).

### 3.4. PTIA and Risk of Mortality

The multivariate modeling for MACCE events within 30 days of surgery showed a positive correlation with age (HR: 1.012 [95% CI: 1.007–1.017], *p* < 0.0001), being female (HR: 1.240 [95% CI: 1.104–1.393], *p* < 0.001), diabetes (HR: 1.231 [95% CI: 1.098–1.381], *p* < 0.001), prior heart failure (HR: 1.516 [95% CI: 1.344–1.709], *p* < 0.0001), and cerebrovascular accidents (HR: 1.488 [95% CI: 1.263–1.754], *p* < 0.0001). The multivariate modeling for all MACCE events anytime during the follow-up period showed similar positive correlations with age (HR: 1.018 [95% CI: 1.016–1.020], *p* < 0.0001), diabetes (HR: 1.386 [95% CI: 1.326–1.449], *p* < 0.001), prior heart failure (HR: 1.551 [95% CI: 1.481–1.626], *p* < 0.0001), and cerebrovascular accidents (HR: 1.552 [95% CI: 1.450–1.660], *p* < 0.0001).

**Figure 2:**
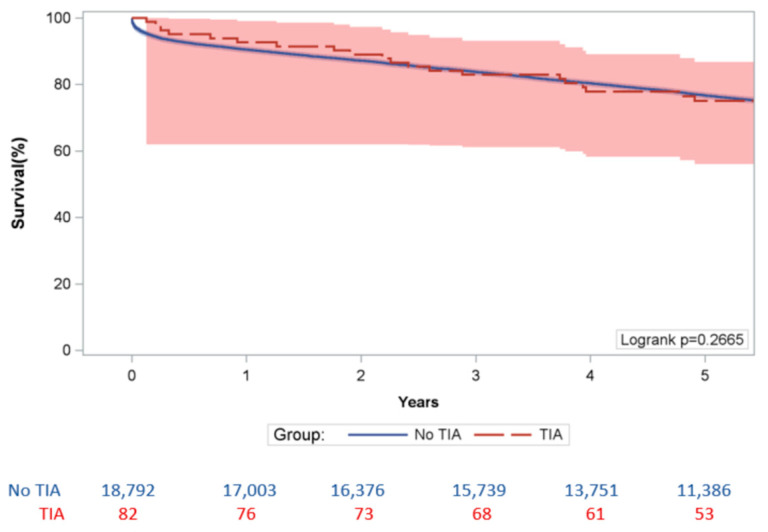
5 Year Kaplan-Meier Survival Analysis.

PTIA did not significantly increase the risk of 30-day MACCE (HR: 0.524 [95% CI: 0.171–1.605], *p* > 0.25) and all MACCE (HR: 1.116 [95% CI: 0.825–1.509], *p* > 0.45) (Table 4). The Kaplan–Meier survival analysis showed the difference in 5-year survival in the “No PTIA” cohort and the “TPIA” cohort were not statistically significant as log rank *p* = 0.2665 (Figure 2).

## 4. Discussion

Based on our results, patients with PTIA have significantly increased key measures of healthcare utilization. This supports research showing that non-operative TIAs cause patients to stay on average 2.6 days longer and cost 40% more than non-affected patients, which is also a concern for PTIAs [9]. Therefore, it is important to evaluate and better define PTIAs, as there is no current standardized clinical definition [10]. Other factors that increased the length of stay following cardiac surgery included being female, diabetes, prior heart failure, and prior cerebrovascular accidents. Interestingly, age was negatively correlated with length of stay. Previous literature has shown that age does not have a monotonous relationship with the length of stay after non-operative TIAs and decreases in certain age groups (50–59 years old); alternatively, it may be attributed to increased rates of death in hospital [11].

PTIA also increased the rates of all-cause readmission, but not stroke readmission. Diabetes and previous cerebrovascular events were correlated with higher rates of stroke and all-cause readmission. In addition, being female and prior heart failure were also risk factors for all-cause readmission. Readmission studies in hospital registries for non-operative TIAs have shown that the most often cause is infection and other non-neurological conditions, which is consistent with our findings [12]. This information can be helpful for hospitals to evaluate readmission reduction strategies in patients with PTIA.

Based on our results, patients who experienced PTIA did not have an increased risk of MACCE and 30-day MACCE. This is interesting given that TIAs that occur in non-operative settings have been shown to significantly increase MACCE and 30-day MACCE events [13]. Our results could be secondary to the low number of PTIAs in the entire cohort and the mortality risk from surgery from other causes. Thus, the impact of PTIA might not be clear as compared to non-operative TIA. The multivariate modeling showed that age, being female, diabetes, prior heart failure, and prior cerebrovascular events were all factors for MACCE events within 30 days of surgery. The modeling for MACCE events at any time during the follow-up showed similar results, with age, diabetes, prior heart failure, and previous cerebrovascular events being positively correlated. This supports the previous literature also showing that long-term MACCE events are correlated with age, previous ischemic strokes, congestive heart failure, GFR < 60 mL/min, mRS ≥ 3 at discharge, and atrial fibrillation [13]. The significantly higher percentage of our study population having long-term MACCE events compared to 30-day MACCE events (41.9% vs. 6.2%, respectively) is similar to previous studies and highlights the need for lifetime follow-up, lifestyle changes, and primary care following cardiac surgery [14].

Our study is unique in that it quantifies PTIA’s impact on outcomes such as MACCE, 30-day MACCE, readmission, and extended length of stay after cardiac surgeries in an extensive academic medical system from a large database. To our knowledge, it is the first study to quantify and report these numbers. The limitations of this study stem from the retrospective nature of the data, the limited sample size of PTIAs in the index population, and the limited patient demographics. Inherent to all large retrospective database studies, it is still unclear what causes PTIA, as our results only measure correlation and not causative factors. We are also limited by a lack of adherence to the proper chart documentation, notably lacking data on PTIA in certain cases. Our model would benefit from more data as our sample size only contains 82 PTIA cases. Our study is also limited by a lack of diversity as our population is predominantly white (92.6%) and male (65.6%) because of only having data from a single regional medical system. Future directions include replicating this study with data from other regions or health systems, utilizing machine learning models or artificial intelligence to create more accurate predictive models, or understanding what factors are causative for PTIA.

## 5. Conclusions

Our results suggest that while PTIA increases risk of ICU stay, hospital length of stay, and all-cause readmission, it does not increase risk of mortality or MACCE events. As a result of PTIA’s significant burden on the healthcare system due to increased utilization, it is critical to better define and recognize PTIA for timely management to improve perioperative outcomes.

## Figures and Tables

**Table 1:** Baseline characteristics.

Index Surgery	Number of Cases	Percentage
Aortic Root	1194	6.33%
CABG and AV	1508	7.99%
CABG and MV	615	3.26%
CABG and PV	2	0.01%
CABG and TV	26	0.14%
Double Valve	1055	5.59%
Isolated AV	2318	12.28%
Isolated CABG	7725	40.93%
Isolated MV	1305	6.91%
Isolated PV	15	0.08%
Isolated TV	111	0.59%
TAVR	1575	8.34%
Triple Valve	96	0.51%
Other	1329	7.04%
Gender		
Female Patients	6500	34.40%
Male Patients	12,374	65.60%
Race		
White Patients	17,474	92.50%
Black Patients	910	4.80%
Other Races	490	2.60%
Comorbidities		
Diabetes	7131	37.70%
Hypertension	15,808	83.70%
COPD	4665	24.72%
Peripheral Vascular Disease	3807	20.17%
Cerebrovascular Disease	4353	23.06%
Previous Myocardial Infarctions	7857	41.60%
Previous Heart Failure	5753	30.48%
Previous CABG	1407	7.45%
Previous Valve Procedure	1365	7.23%
Dialysis	520	2.76%
Biodata	Median	q1–q3
Median Age (years)	68	59–76
Body Mass Index	28.73	25.26–33.10
Last Creatine Level	1	0.8–1.2
Cross Clamp Time (min)	86	62.00–121.00
Cardiopulmonary Bypass Time (min)	116	87.00–158.00

**Table 2:** Multivariable model for longer hospitalization.

Variable	HR (95% CI)	*p*-Value
PTIA	2.199 (1.416–3.416)	<0.001
Female	1.216 (1.141–1.296)	<0.001
Diabetes	1.267 (1.191–1.349)	<0.001
Prior Heart Failure	1.421 (1.330–1.518)	<0.0001
Cerebrovascular Accidents	1.695 (1.534–1.873)	<0.0001
Age	0.993 (0.990–0.995)	<0.0001

**Table 3:** Fine and gray modeling for readmission.

Variable	Stroke Readmission HR (95% CI)	*p*-Value	All-Cause Readmission HR (95% CI)	*p*-Value
Diabetes	1.205 (1.091–1.330)	<0.001	1.248 (1.200–1.298)	<0.0001
Cerebrovascular Accidents	1.772 (1.545–2.033)	<0.001	1.196 (1.121–1.77)	<0.0001
PTIA	1.643 (0.913–2.956)	<0.10	1.444 (1.096–1.902)	<0.01
Age	1.003 (0.999–1.007)	<0.15	1.001 (0.999–1.002)	<0.45
Female	1.018 (0.919–1.128)	<0.75	1.077 (1.034–1.122)	<0.0001
Prior Heart Failure	1.065 (0.956–1.186)	<0.30	1.279 (1.226–1.334)	<0.0001

**Table 4:** 30-day MACCE and all MACCE.

Variable	30-Day MACCE HR (95% CI)	*p*-Value	All MACCE HR (95% CI)	*p*-Value
Age	1.012 (1.007–1.017)	<0.0001	1.018 (1.016–1.020)	<0.0001
Female	1.240 (1.104–1.393)	<0.001	1.031 (0.985–1.080)	>0.19
Diabetes	1.231 (1.098–1.381)	<0.001	1.386 (1.326–1.449)	<0.001
Prior Heart Failure	1.516 (1.344–1.709)	<0.0001	1.551 (1.481–1.626)	<0.0001
Cerebrovascular Accidents	1.488 (1.263–1.754)	<0.0001	1.552 (1.450–1.660)	<0.0001
PTIA	0.524 (0.171–1.605)	>0.25	1.116 (0.825–1.509)	>0.45

## Data Availability

Data is unavailable due to privacy and ethical restrictions. The authors had full access to all the data in the study and take responsibility for the integrity of the data and the accuracy of the data analysis.
